# Coronary Artery Bypass Graft Surgery Cost Coverage by the Brazilian Unified Health System (SUS)

**DOI:** 10.21470/1678-9741-2016-0069

**Published:** 2017

**Authors:** Gilmara Silveira da Silva, Flávia Cortez Colósimo, Alexandre Gonçalves de Sousa, Raquel Ferrari Piotto, Valéria Castilho

**Affiliations:** 1 Real e Benemérita Associação Portuguesa de Beneficência (Hospital Beneficência Portuguesa de São Paulo), São Paulo, SP, Brazil; 2 Escola de Enfermagem da Universidade de São Paulo (EE-USP), São Paulo, SP, Brazil

**Keywords:** Health care costs, Hospitalization, Coronary Artery Bypass, Unified Health System, Nursing Administration Research

## Abstract

**Introduction:**

Cost management has been identified as an essential tool for the general control and evaluation of health organizations.

**Objectives:**

To identify the coverage percentage of transferred funds from the Unified Health System for coronary artery bypass grafts in a philanthropic hospital having a consolidated costing system in the municipality of São Paulo.

**Methods:**

A quantitative, descriptive and cross-sectional research with information provided from a database composed of 1913 patients undergoing coronary artery bypass graft from March 13 to September 30, 2012, including isolated elective coronary artery bypass graft with the use of extracorporeal circulation. It excluded 551 (28.8%) patients, among them 76 (4.0%) deaths and 8 hospitalized patients, since the cost was compared according to the length of hospital stay. Therefore, the sample consisted of 1362 patients.

**Results:**

The average total cost per patient was $7,992.55. The average fund transfer by the Unified Health System was $3,450.73 (48.66%), resulting in a deficit of $4,541.82 (51.34%).

**Conclusion:**

The Unified Health System transfers covered 48.66% of the average total cost of hospitalization. Although the amount transferred increased with increasing costs, it was not proportional to the total cost, resulting in a percentage difference in revenue that was increasingly negative for each increase in cost and hospital stay. Those hospitalized for longer than seven days presented higher costs, older age, higher percentage of diabetics and chronic kidney disease patients and more postoperative complications.

**Table:** 

Abbreviations, acronyms & symbols	
CABG	= Coronary artery bypass graft
EC	= Extracorporeal circulation
ICU	= Intensive care unit
IHA	= Inpatient hospital authorization
LOS	= Length of stay
MOH	= Ministry of Health
SUS	= Unified Health System

## INTRODUCTION

The Brazilian health system is undergoing an important financial crisis that threatens the survival of hospital organizations, especially philanthropic ones, that play an essential role in serving the poorest class of the population.

These philanthropic institutions benefit from tax immunity, but must allocate 60% of their capacity to the Unified Public Health System (SUS) or 20% of their total services as free services to the population^[1-3]^. In this way, they face serious financial problems that may compromise their survival^[4]^ and impact on meeting the population's demand, considering the various health care procedures that are performed by SUS throughout Brazil. Therefore, cost management has been pointed out as an essential tool for controlling and evaluating these organizations, both public and private^[4,5]^.

Among the several cardiac surgeries performed by SUS in Brazil, the most frequent is coronary artery bypass graft (CABG), performed in both public and private hospitals throughout the country^[4]^. However, few Brazilian studies have analyzed the cost of this complex procedure and the specific transfer of funds from SUS regarding this surgery.

Cost is defined as an expense relating to a good or service used in the production of other goods or services, and may be classified into direct, indirect, fixed or variable cost, and the sum of all these cost forms is defined as total cost^[6]^. The most widely used costing system by hospitals to gauge total costs is known as the Absorption Costing System. The main characteristics of this costing system are the need for apportionment in the case of appropriation of indirect costs, and that the results are directly influenced by the production volume^[6]^.

On the other hand, the transfer of funds from SUS consists of the amount of money paid by the Ministry of Health (MOH) to health institutions for their performed procedures, pursuant to Administrative Rule no. 204 of January 29, 2007, which regulates the financing and transfer of federal resources to health actions and services, with appropriate monitoring and control^[7]^.

SUS spent more than R$209 million to perform isolated CABGs and/or those associated with other procedures in the city of São Paulo from 2011 to 2014, which corresponded to an average expenditure of R$12,025.66 per patient^[8]^. These expenses are derived from amounts transferred from SUS measuring or from contracts that some hospitals establish with the system, and not by measuring the costs of surgeries.

Thus, it is unknown how this total cost is conferred in the different hospitals in Brazil and if the fund transfer from SUS can cover them.

Therefore, the purpose of this study is to identify the coverage percentage of hospitalization costs for CABG by fund transfer from SUS in a philanthropic hospital in the city of São Paulo that implements the consolidated Absorption Costing System.

## METHODS

A quantitative, descriptive and cross-sectional study carried out in the Beneficência Portuguesa Hospital of São Paulo, which is a philanthropic, special-purpose hospital in the city of São Paulo. This hospital has a database started in 2009, which collects information on their performed myocardial revascularization surgeries such as demography, diagnostic exams, surgical techniques, complications, mortality, drug treatment, length of hospital stay, and costs, among others, and is part of the continuous improvement of the institution's care practices.

The information for this study was extracted from a database composed of 1913 patients submitted to CABG at the surveyed institution, aged 18 years or older in the period from March 13 to September 30, 2012, and includes data from 100% of all CABG performed at the hospital in this period.

The choice for this period was due to this being the beginning of information inclusion on cost and fund transfer from SUS available for study. The database has 1913 patients. Isolated surgeries (without other associated procedures), elective surgeries (which were not urgent or an emergency), surgeries using extracorporeal circulation (EC) and those that had SUS funding as a source for hospitalization costs were included in the study. Five hundred and fifty-one (551-28.8%) patients were excluded, totaling 1362 for the study sample. However, the sum of exclusion was not 551, since some patients were excluded due to more than one reason for not participating in the study, according to the exclusion criteria [172 (9%) non-SUS; 13 (0.7%) for not having cost information; 76 (4.0%) deaths, 206 (10.8%) non-isolated CABG; 17 (0.9%) urgencies; 1 (0.05%) emergency; 132 (6.9%) without EC and 8 (0.4%) patients who remained in the ICU at the time of the database closing on the 30^th^ postoperative day]. Deaths and inpatients were excluded since the evaluated cost refers to hospital admission, and groups were compared according to length of hospital stay.

The research was conducted in accordance with Resolution of the National Health Council, number 466 of December 12, 2012, and authorized by the Research Ethics Committees of the Nursing School of the University of São Paulo, with Opinion No: 1.024.151 on 4/15/15, and of the institution under study with Opinion No: 1.088,381 on 06/01/15. Patient identification data were not made available. Clear and informed consent forms were not solicited from patients since this is a retrospective and documentary study.

### Surveying Costs and Measuring Revenues

We analyzed the cost variables available in the database such as the total cost of hospital admissions, revenue transfers by SUS and the difference between revenue and cost. The Brazilian real (R$) was converted to US dollar ($). We used the quotation value of the dollar (purchase) on 09/28/2012, which was 2.0265 reais^[9]^. We considered the dollar value close to the finishing date of data collection (09/30/2012), since there was no access to the cost per patient at the time of hospital discharge.

The Absorption Costing System was implemented in 2010 into the hospital under study from the perspective of managerial accounting with the formation of cost centers. Thus, it was possible to gauge total costs by summing the direct, indirect, fixed and variable costs of the services, procedures and examinations, transposing the values into daily hospital charges and fees.

The fund transfer process from SUS is done according to inpatient hospital authorizations (IHA)^[8]^ generated for the patient. The Beneficência Portuguesa Hospital uses a consolidated controlling service from SUS, which sends the debits from specific sites to MOH, which are analyzed, and the corresponding values are then transferred to the hospital.

### Data Analysis

All variables were initially analyzed descriptively. Minimum and maximum values were analyzed for variable cost, revenue and time intervals, and averages, standard deviations and medians were then calculated. Absolute and relative frequencies were calculated for the group variables of hospitalization duration and patients' profile characterization. Non-parametric Mann-Whitney test was used for comparison of length of hospitalization groups in relation to cost and income variables^[10]^. Chi-square test^[10]^ or Fisher's exact test^[10]^ (for expected frequencies lower than 5) were used in order to test homogeneity among the proportions of hospitalization groups. The level of significance used for the tests was 5%.

## RESULTS

The results are presented in three parts. The first part presents a panoramic view of data on costs, revenues and funds transferred from SUS for CABG hospitalization (Table 1). The second part shows the average coverage percentage of funds transferred for the average total cost of hospitalization per patient (Table 2) and seeks to compare the average coverage percentage among groups according to the length of hospital stay (Table 3). The third part compares these two groups in terms of age, gender, EuroSCORE I^[11]^, preoperative risk factors and postoperative complications (Table 4). Cost data are presented in American dollars^[9]^.

**Table 1:** Comparison of the descriptive values of cost, revenue and percentage of funds transferred from SUS in American dollars ($), for patients undergoing coronary artery bypass surgery from March to September 2012 - São Paulo, Brazil, 2017.

Patients	n	Average total cost $	Direct cost $	Indirect cost $	Average revenue $	Difference cost x revenue $	Revenue deficit %	Funding coverage %
Consolidated total	1362	10,885,855.91	6,313,796.43	4,898,635.16	4,699,897.85	-6,185,958.06	-56,83^(1)^	43,17^(2)^
Total/patient	1362	7,992.55	4,635.68	3.356.87	3,450.73	-4,541.82	-51,34^(1)^	48,66^(2)^
Readmitted to Intensive Care Unit	156	14,588.02	8,461.06	6.126.97	4,847.57	-9,740.44	-58,49^(1)^	41,51^(2)^
Length of stay ≤ 7 days	416	4,978.73	2,887.66	2.091.07	2,835.27	-2,143.46	-41,91^(1)^	58,09^(2)^
Length of stay > 7 days	946	8,926.42	5,177.33	3.749.10	3,721.38	-5,596.50	-55,48^(1)^	44,52^(2)^

Descriptive level of probability of the non-parametric Mann-Whitney test.

(1) Percentage individually calculated through statistical analysis and with the value rounded for each patient in the sample (descriptive level of probability of the non-parametric Mann Whitney test).

(2) Transfer coverage calculated by the direct average calculation, based on the value of the difference in revenue deficit.The quotation of the US dollar (purchase) on 09/28/2012 was 2.0265 reais (http://economia.uol.com.br/cotacoes/cambio/dolarcomercial-estados-unidos/?historico).

**Table 2:** Cost and revenue descriptive values in American dollars ($), for patients submitted to coronary artery bypass surgery from March to September 2012 - São Paulo, Brazil, 2017 (n=1362).

Variable	Mean $	SD $	Median $	Minimum $	Maximum $
Cost	7,992.55	7,724.18	6,462.84	3,300.26	152,132.10
Revenue	3,450.73	1,774.08	3,159.37	2,523.79	28,874.10
Difference	-4,541.82	6,295.88	-3,303.64	-124,745.13	2,095.49
Difference %	-51.34	10.81	-50.88	-89.00	19.36

Percentage individually calculated through statistical analysis and with the value rounded for each patient in the sample. The quotation of the US dollar (purchase) on 09/28/2012 was 2.0265 reais (http://economia.uol.com.br/cotacoes/cambio/dolar-comercial-estados-unidos/?historico)

**Table 3:** Comparison of the descriptive values of cost and revenue in American dollars ($), for patients undergoing coronary artery bypass surgery from March to September 2012, according to the length of stay - São Paulo, Brazil, 2017.

Variable	Stay	N	Mean $	SD $	Median $	Minimum $	Maximum $	*P*
Cost	≤7 days	416	4,978.73	1,109.62	4,834.28	3,300.26	20,654.31	<0.001
	>7 days	946	9,317.87	8,923.55	7,319.40	3,750.24	152,132.10	
Revenue	≤7 days	416	2,835.27	317.05	2,795.56	2,523.79	6,036.61	<0.001
	>7 days	946	3,721.38	2,061.22	3,276.03	2,526.93	28,874.10	
Difference	≤7 days	416	-2,143.46	954.94	-2,031.52	-15,728.35	453,78	<0.001
	>7 days	946	-5,596.50	7,282.94	-4,011.12	-124,745.13	2,095.49	
Difference %	≤7 days	416	-41.91	7.54	-42.37	-76.15	8.13	<0.001
	>7 days	946	-55.48	9.32	-54.67	-89.00	19.36	

Percentage individually calculated through statistical analysis and with the value rounded for each patient in the sample (descriptive level of probability of the non-parametric Mann Whitney test). The quotation of the US dollar (purchase) on 09/28/2012 was 2.0265 reais (http://economia.uol.com.br/cotacoes/cambio/dolar-comercial-estados-unidos/?historico).

**Table 4:** Comparison of the population profile, EuroSCORE, risk factors and postoperative complications of patients submitted to coronary artery bypass surgery from March to September 2012, according to the length of hospital stay - São Paulo, 2017.

Variable		Duration		*P*
		≤ 7 days (n=416)	> 7 days (n=946)	
Age (in years) (mean + SD)		60.6 ± 8.6	61.8±8.9	0.018^(1)^
Males (n, %)		296 (71.2)	656 (69.3)	0.503^(2)^
EuroSCORE > 5 (n, %)		30 (7.2)	72 (7.6)	0.796^(2)^
Risk factors (n, %)	Systemic arterial hypertension	373 (89.7)	859 (90.8)	0.510^(2)^
	Diabetes	135 (32.5)	406 (42.9)	<0.001^(2)^
	Dyslipidemia	226 (54.3)	522 (55.2)	0.771^(2)^
	Chronic kidney failure	9 (2.2)	58 (6.1)	0.002^(2)^
	Previous cerebrovascular accident	13 (3.1)	51 (5.4)	0.069^(2)^
	Arrhythmia	8 (1.9)	26 (2.8)	0.369^(2)^
	Prior/chronic atrial fibrillation	4 (1.0)	15 (1.6)	0.366^(2)^
Complication (n, %)	Transfusion	140 (33.7)	503 (53.2)	<0.001^(2)^
	Atrial fibrillation requiring intervention	24 (5.8)	207 (21.9)	<0.001^(2)^
	Major bleeding	43 (10.3)	160 (16.9)	0.002^(2)^
	Pneumonia	0 (0.0)	71 (7.5)	<0.001^(2)^
	Acute renal failure	2 (0.5)	43 (4.6)	<0.001^(2)^
	Perioperative acute myocardial infarction	3 (0.7)	24 (2.5)	0.027^(2)^
	Dialysis	0 (0.0)	16 (1.7)	0.005^(3)^
	Cerebrovascular accident	0 (0.0)	16 (1.7)	0.005^(3)^
	Prolonged mechanical ventilation	0 (0.0)	16 (1.7)	0.005^(3)^
	Reoperation for bleeding	0 (0.0)	6 (0.6)	0.186^(3)^
	Reoperation for bleeding/mediastinitis	0 (0.0)	10 (1.1)	0.037^(3)^
	Reoperation for mediastinitis	0 (0.0)	4 (0.4)	0.320^(3)^
	Mediastinitis with clinical treatment	0 (0.0)	6 (0.6)	0.186^(3)^

(1) Descriptive probability level Student's t-test.

(2) Descriptive probability level Chi-square test.

(3) Descriptive probability level Fisher's exact test.

### Coverage Percentage of Funds Transferred from SUS in Hospital Admission for CABGs

According to Table 1, the consolidated total cost on hospital admission for the sample of 1362 patients was $10,885,855.91, and consolidated revenue was $4,699,897.85, with the total funds transferred from SUS being 43.17% of the costs, and total negative revenue of 56.83%.

The total average cost of hospitalization per patient as described in Table 1 was $7,992.55, and according to information provided by the Hospital Cost Management under study, the direct cost of hospitalization for CABGs in 2012 was 58% of the total cost, $4,635.68, and the indirect cost was 42%, $3,356.87.

According to Table 1, the average revenue transferred by SUS was $3,450.73, presenting an average coverage percentage of 48.66% of the total cost, thus generating average negative revenue of 51.34% to the hospital, of which the largest portion is destined to the payment of medical fees.

A difference was found in relation to the average revenue deficit and the average of SUS transfers shown in Table 1, since the average revenue deficit and all calculations of cost, revenue and the percentage difference between cost and revenue were statistically and individually calculated for each of the 1362 patients in the sample. The values were rounded for each patient with a difference or error occurring when comparing the direct total average calculation as calculated by SUS transfers in the last column of the table. This calculation of the transfer percentage was based on the percentage difference of the revenue deficit.

According to Table 2, the total cost ranged from $3,300.26 to $152,132.10. Of these, 22 (1.62%) cases had a total cost between $49,346.16 and $98,692.33, four (0.29%) cases between $98,692.33 and $148,038.49 and one (0.07%) case above $148,038.49.

Data from Table 1 also show intensive care unit (ICU) readmission occurred in 156 (11.5%) patients. Comparing the total cost and the funds transferred from SUS among readmitted and non-readmitted patients (1206), it was verified that although those readmitted had higher costs and higher revenues, the difference between cost and revenue was higher, thus generating a higher percentage of negative revenue in relation to the group without readmission (58.49% *vs.* 50.41%, *P*<0.001). Therefore, the average coverage percentage of transferred funds was 41.51% for readmitted patients and 49.59% for non-readmitted patients.

### Mean Percentage of Hospital Admission Coverage in CABGs and Length of Stay (LOS)

Average LOS was 11.23 days (2.73 preoperative, 8.50 postoperative, of which 2.42 were in ICU). At the institution under study, the medical practice manual defines that LOS in isolated and elective CABGs should be up to seven days.

Thus, for a better cost evaluation and fund transfer from SUS, patients were divided into Group 1 (LOS≤7 days) and Group 2 (LOS>7 days), and in comparing the groups, it was possible to verify that 69.5% of the sample presented LOS>7 days.

It can be observed in Table 3 that Group 2 presents significantly higher cost, revenue, difference between cost-revenue and percentage difference (*P*<0.001).

According to Table 1, Group 2 presented a coverage percentage from SUS lower than Group 1 with 44.52% *versus* 58.09%, respectively, generating a significantly higher percentage of negative revenue in the group of patients who spent more than seven days hospitalized.

### Comparison of Groups in Relation to Preoperative and Postoperative Profile

Of the 1362 patients, the mean age was 61.4 years and 69.9% were males. In attempt to justify the increase in the hospital stay and costs, the groups' preoperative profiles were compared. According to Table 4, the expected mortality risk assessed by EuroSCORE I^[11]^ did not differ, but in relation to the preoperative factors, Group 2 had a higher age (*P*=0.018), a higher number of diabetics (*P*<0.001) and a higher number of patients with chronic kidney disease (*P*=0.002).

Regarding the presence of postoperative complications, Table 4 shows that Group 2 presented a higher need for blood transfusion, atrial fibrillation requiring intervention, major bleeding, pneumonia, acute renal failure, acute perioperative myocardial infarction, hemodialysis, stroke, prolonged mechanical ventilation (up to 48 hours) and reoperation due to bleeding and/or mediastinitis.

## DISCUSSION

Data from the present research showed that the total funds transferred from SUS only covered 43.17% of the total consolidated cost and generated a revenue deficit of 56.83%. The funds transferred per patient were only 48.66%, lower than the direct cost of hospitalization.

Although CABG is the most performed heart surgery by SUS in Brazil, few studies have analyzed the costs of the procedure. This lack of literature may be due to the fact that health institutions do not have a Consolidated Costing System.

In the few Brazilian studies on CABG costs, it can be noticed that some did not assess procedure nor hospitalization costs. Some authors have considered the values of fixed tables as costs based on Inpatient Hospital Authorization (IHA) or on prices of pre-established packages^[4,12]^. Others cited the transferred funds from SUS as a direct cost and additional institutional costs as indirect costs^[13]^, which may underestimate the real cost of the procedure.

A study carried out in a public hospital in the city of São Paulo revealed that coverage from SUS for the total average cost of the procedure in primary, isolated and elective CABGs in 2005 was 79.41% of the direct costs of the procedure^[14]^. However, the total cost of hospitalization was not included, which could significantly reduce this percentage.

According to the Health Information Portal of the Municipality of São Paulo/IHA, the consolidated funds transferred from SUS through the total amount of IHA paid for CABG (isolated and associated with other procedures) from 2011 to 2014 was R$209,883,898.93; of which R$57,094,462.41 in 2011, R$55,322,437.14 in 2012, R$48,746,376.00 in 2013 and R$48,720,623.38 in 2014. However, the average transferred per patient from 2011 to 2014 was R$12,025.66, with R$11,862.55 in 2011, R$12,052.82 in 2012, R$12,045.06 in 2013 and R$12,171.03 in 2014^[8]^.

Comparing the consolidated funds transferred from SUS for CABGs of the present research to the municipality of São Paulo in 2012^[8]^, it was verified that the value transferred to the institution under study corresponded to 17.22% of the funds transferred to the municipality. It is worth mentioning that the research institution was responsible for 16.4 to 17.5% of the CABGs performed by SUS in the period from 2008 to 2014^[15,16]^, a significant percentage of this procedure in the country.

When comparing the mean of funds transferred from SUS for CABG per patient in 2012 between data from this research and data from the city of São Paulo^[8]^, it was verified that the funds transferred in the present research corresponded to 58.02% of the average per patient in the municipality. However, the study sample was from isolated and elective CABGs. This shows that these transferred funds may have increased according to the patient's severity, as demonstrated by authors in 2015^[17]^.

Most Brazilian studies only address the direct cost of the procedure, with almost no studies addressing the total cost or transferred funds from SUS for CABGs. In 2015, a study was published on average total cost and transfers from SUS for cardiac surgeries performed in 2013, with 48.8% of the evaluated surgeries being CABGs^[17]^. This study showed that transfers increased according to the severity of the EuroSCORE^[11]^, while the funds transferred for low-risk patients was R$14,306.00, for medium-risk R$16,217.00, and R$19,548.00 for high-risk patients (*P*<0.001)^[17]^, corresponding to coverages of 52.75%, 46.53% and 45.21%, respectively. The authors demonstrated that the transferred funds were not proportional to the total cost, presenting a lower coverage percentage as the severity, cost and LOS increased.

The average LOS in this study was 11.23 days, which was below the average of São Paulo in 2012 (13.29 days)^[8]^. However, prolonged stay (LOS>7days) occurred in 69.5% of the patients.

Another study on isolated CABGs and those associated with other procedures in 2010 in the same institution under study found a mean LOS of 11 days, with prolonged stay in 62.67% of the patients^[18]^.

In these two years (2010 and 2012), there was no significant difference in the mean of LOS, but the percentage of prolonged stay increased by 6.83%, thus making a more detailed study of this population very interesting.

Comparing the total average cost of hospitalization between groups according to the LOS, there was a significant increase in the cost for Group 2 (group with LOS>7 days), (*P*<0.001), and although the transferred funds were greater for this group, it was noticed that Group 1 (group with LOS less than or equal to 7 days) had a higher coverage percentage of the total average cost (Group 1 had 58.09% *vs.* Group 2 with 44.52%).

Therefore, this demonstrates that the higher the LOS, the higher the cost and the funds transferred from SUS (*P*<0.001). However, the funds transferred were not proportional to the cost, thereby resulting in an increasingly negative percentage difference of revenue for each increase in cost and hospital stay.

When comparing the average coverage of the funds transferred per patient in the present study with that of a study published in 2013^[17]^, the coverage was around 45 to 52%. In the present study, the coverage was 48.66% of the costs. In the cited study^[17]^, the coverage in increasing order of mortality risk by the additive EuroSCORE (low, medium and high)^[11]^ was 52.75%, 46.53% and 45.21%, respectively. In both works, an increase in hospitalization increased the cost and the value of funds transferred from SUS; however, the increase in the transferred funds did not proportionately follow the increase in total cost, with the coverage percentage decreasing at each increase in LOS and cost.

CABG costs have increased over time due to technological advances. However, even though the funds transferred from SUS have increased, they remain insufficient and cover just about half of hospital costs. This mismatch between cost and funds being transferred can cause financial losses to healthcare institutions, which are contracted with SUS throughout the country, compromising their market survival and their ability to meet the population's demand. This characterizes the problem of the arduous financial question inherent in health institutions to accomplish this complex procedure.

The data of this study corroborate the affirmation of some authors that, in relation to the destination of government funds for public health expenditures, the transfer of funds from SUS is out of date and insufficient to cover the costs of performed surgical procedures, and this scenario may worsen with the increase in the number of cardiovascular surgeries^[4,17]^.

In view of this, the question arises as to the need to seek strategies for managing and reducing costs, such as reducing LOS, increasing bed rotation and increasing attending demand, in line with SUS principles of accessibility for patients. It is necessary to review health policies in government offices for contracting the value of the funds transferred from SUS, ensuring the financial integrity of hospital institutions.

When comparing the groups' preoperative profile according to LOS in an attempt to justify the increase in hospital stay and cost, the mortality risk expected by the EuroSCORE calculation^[11]^ did not differ between groups. However, in relation to the preoperative factors, Group 2 had a higher age, a greater number of diabetics, and chronic kidney disease patients. Data from a Brazilian study also identified the presence of diabetes and kidney disease, among others, as factors that increased hospital stay^[19]^.

Group 2 presented the highest number of postoperative complications, which shows that the presence of complications is related to the increase in hospital stay, corroborating data from a Brazilian study^[19]^ and the cost. An American study of 65,534 patients analyzed the cost of postoperative morbidity from cardiac surgeries performed from 2001 to 2011, and concluded that postoperative complications impact increased hospital stay and costs^[20]^. Other authors have also stated that prolonged postoperative hospitalization may contribute to increased severity, reduction of functional capacity and productivity^[19,21,22]^, affect family budgets, the patient's quality of life and generate an increase in public spending in general^[23]^.

Group 2 patients, although not more severe according to the EuroSCORE, remained longer than the protocol of the institution under study. However, this considered increased hospitalization is related to the presence of more advanced age, comorbidities such as diabetes and chronic kidney disease, and a greater number of postoperative complications; a finding similar to that presented by other studies^[19,24]^.

New Brazilian studies on the coverage of funds transferred from SUS for the total hospitalization costs in elective isolated CABGs will be necessary for a real comparability with the data of this research, and may contribute to having more parameters on health costs in Brazil.

### Limitations of the Study

Since this is a study with a database, only three cost variables (total cost, funds transferred by SUS and the difference between revenue and cost) were found.

Also, information on direct and indirect, fixed and variable costs of the surgical procedure itself and of each cost center used in patient care were not found.

## CONCLUSION

Funds transferred from SUS covered less than half the total average hospitalization costs of CABGs (48.66%). Although the funds transferred from SUS increased according to costs, these transfers were not proportional to the total cost, resulting in a percentage difference of increasingly negative revenue with each increase in cost and hospital stay. The increase in the hospital stay (considered to be prolonged when LOS is over 7 days) increased the cost, and this group had older patients with a higher percentage of diabetic and chronic kidney disease patients, and more postoperative complications.

**Table:** 

Authors' roles & responsibilities	
GSS	Analysis and/or data interpretation; conception and design study; manuscript redaction or critical review of its content; statistical analysis; final manuscript approval
FCC	Analysis and/or data interpretation; conception and design study
AGS	Analysis and/or data interpretation; conception and design study;
RFP	Analysis and/or data interpretation; conception and design study; manuscript redaction or critical review of its content; statistical analysis; final manuscript approval
VC	Manuscript redaction or critical review of its content; final manuscript approval

## References

[1] Brasil (2002). Decreto n. 4.327, de 8 de agosto de 2002. Aprova critérios
para concessão do certificado de filantropia para os
hospitais. Diário Oficial da União.

[2] Portela MC, Lima SM, Barbosa PR, Vasconcellos MM, Ugá MA, Gerschman S (2004). Characterization of assistance among philanthropic hospitals in
Brazil. Rev Saude Publica.

[3] Lima SM, Portela MC, Ugá MA, Barbosa PR, Gerschman S, Vasconcellos MM (2007). Philanthropic hospitals and the operation of provider-owned
health plans in Brazil. Rev Saude Publica.

[4] Piegas LS, Bittar OJ, Haddad N (2009). Myocardial revascularization surgery (MRS): results from National
Health System (SUS). Arq Bras Cardiol.

[5] Castilho V, Lima AF, Fugulin FM, Peres HH, Gaidzinski RR (2014). Total staff costs to implement a decision support system in
nursing. Rev Latino Am Enfermagem.

[6] Martins E (2009). Contabilidade de custos.

[7] Brasil, Ministério da Saúde (2007). Regulamenta o financiamento e a transferência de recursos
federais para as ações e os serviços de saúde,
na forma de blocos de financiamento, com o respectivo monitoramento e
controle. Portaria n. 204, de 29 de janeiro de 2007.

[8] Brasil, Ministério da Saúde, DATASUS (2015). AIH pagas por ano de competência em cirurgia de
revascularização miocárdica com uso de
circulação extracorpórea. Internações hospitalares do SUS no Município de
São Paulo a partir de 2008.

[9] (2017). Economy: Quotations.

[10] Rosner B (1986). Fundamentals of biostatistics.

[11] Nashef SA, Roques F, Michel P, Gauducheau E, Lemeshow S, Salamon R (1999). European system for cardiac operative risk evaluation
(EuroSCORE). Eur J Cardiothorac Surg.

[12] Almeida RMS (2005). Myocardial revascularization: comparative cost study between
conventional coronary bypass and percutaneous transluminal coronary
angioplasty. Braz J Cardiovasc Surg.

[13] Girardi PB, Hueb W, Nogueira CR, Takiuti ME, Nakano T, Garzillo CL (2008). Comparative costs between myocardial revascularization with or
without extracorporeal circulation. Arq Bras Cardiol.

[14] Haddad N, Bittar E, Marchi AF, Kantorowitz CS, Ayoub AC, Fonseca ML (2007). Hospital costs of coronary artery bypass grafting on elective
coronary patients. Arq Bras Cardiol.

[15] Brasil, Ministério da Saúde, DATASUS (2014). Sistema de Informações Hospitalares. Programa
TabWin.

[16] Brasil, Ministério da Saúde, DATASUS (2016). Procedimentos hospitalares do SUS - por local de
internação - Brasil.

[17] Titinger DP, Lisboa LA, Matrangolo BL, Dallan LR, Dallan LA, Trindade EM (2015). Cardiac surgery costs according to the preoperative risk in the
Brazilian public health system. Arq Bras Cardiol.

[18] Silva GS, Sousa AG, Soares D, Colósimo FC, Piotto RF (2013). Evaluation of the length of hospital stay in cases of coronary
artery bypass graft by payer. Rev Assoc Med Bras.

[19] Laizo A, Delgado FE, Rocha GM (2010). Complications that increase the time of hospitalization at ICU of
patients submitted to cardiac surgery. Rev Bras Cir Cardiovasc.

[20] LaPar DJ, Crosby IK, Rich JB, Fonner E Jr, Kron IL, Ailawadi G, Investigators for Virginia Cardiac Surgery Quality
Initiative (2013). A contemporary cost analysis of postoperative morbidity after
coronary artery bypass grafting with and without concomitant aortic valve
replacement to improve patient quality and cost-effective
care. Ann Thorac Surg.

[21] Oliveira EK, Silva VZ, Turquetto AL (2009). Relationship on walk test and pulmonary function tests with the
length of hospitalization in cardiac surgery patients. Rev Bras Cir Cardiovasc.

[22] Atoui R, Ma F, Langlois Y, Morin J (2008). Risk factors for prolonged stay in the intensive care unit and on
the ward after cardiac surgery. J Card Surg.

[23] Lloyd-Jones D, Adams R, Carnethon M, De Simone G, Ferguson TB, Flegal K, American Heart Association Statistics Committee and Stroke
Statistics Subcommittee (2009). Heart disease and stroke statistics--2009 update: a report from
the American Heart Association Statistics Committee and Stroke Statistics
Subcommittee. Circulation.

[24] Fernandes MVB, Aliti G, Souza EN (2009). Profile of patients undergoing to coronary artery bypass
grafting: implications for nursing care. Rev Eletr Enf.

